# Metabolomic analysis of fibrotic mice combined with public RNA‐Seq human lung data reveal potential diagnostic biomarker candidates for lung fibrosis

**DOI:** 10.1002/2211-5463.12982

**Published:** 2020-10-05

**Authors:** Yosui Nojima, Yoshito Takeda, Yohei Maeda, Takeshi Bamba, Eiichiro Fukusaki, Mari N. Itoh, Kenji Mizuguchi, Atsushi Kumanogoh

**Affiliations:** ^1^ Laboratory of Bioinformatics Artificial Intelligence Center for Health and Biomedical Research (ArCHER) National Institutes of Biomedical Innovation, Health and Nutrition Osaka Japan; ^2^ Department of Respiratory Medicine and Clinical Immunology Osaka University Graduate School of Medicine Japan; ^3^ Department of Biotechnology Graduate School of Engineering Osaka University Japan; ^4^ Division of Metabolomics Medical Institute of Bioregulation Kyushu University Fukuoka Japan; ^5^ Institute for Protein Research Osaka University Japan

**Keywords:** BALF, biomarker, fibrotic mouse, IPF, metabolomics, serum

## Abstract

Idiopathic pulmonary fibrosis (IPF) is a severe lung disease with poor survival that warrants early and precise diagnosis for timely therapeutic intervention. Despite accumulating genomic, transcriptomic, proteomic, and lipidomic data on IPF, evidence from water‐soluble metabolomics is limited. To identify biomarkers for IPF from water‐soluble metabolomic data, we measured the levels of various metabolites in bronchoalveolar lavage fluid (BALF) and serum samples from a bleomycin‐induced murine pulmonary fibrotic model using gas chromatography/mass spectrometry. Thirty‐two of 73 BALF metabolites and 29 of 74 serum metabolites were annotated. We observed that the levels of proline and methionine were higher in BALF but lower in serum than those in the control. Furthermore, analysis of public RNA‐Seq data from the lungs of patients with IPF revealed that proline‐ and methionine‐related genes were significantly upregulated compared to those in the lungs of healthy controls. These results suggest that proline and methionine may be potential biomarkers for IPF and may help to deepen our understanding of the pathophysiology of the disease. Based on our results, we propose a model capable of recapitulating the proline and methionine metabolism of fibrotic lungs, thereby providing better means for studying the disease and developing novel therapeutic strategies for IPF.

## Graphical abstract

Metabolomic analysis in fibrotic mouse models revealed high levels of proline and methionine in bronchoalveolar lavage fluid but low levels in the serum. Analysis of public RNA‐Seq data from idiopathic pulmonary fibrosis (IPF) patient lungs showed a high expression of proline‐ and methionine‐related genes. The metabolic network generated by the integration of the two omics data suggests proline and methionine as potential biomarkers for IPF.

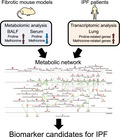

**Abbreviations**
ARG2: arginase 2
BALF: bronchoalveolar lavage fluid
IPF: idiopathic pulmonary fibrosis
OAT: ornithine aminotransferase
P4H: prolyl 4‐hydroxylase
PCA: principal component analysis
TPM: transcripts per million

Idiopathic pulmonary fibrosis (IPF) is a progressive lung disease, and its diagnosis and management remain challenging [1, 2]. It is believed that alveolar epithelial damage, and subsequent dysregulated wound repair, may lead to progressive pulmonary fibrosis [3, 4]. Factors that damage the epithelium such as smoking, wood dust, and epithelial endoplasmic reticulum stress have been linked to IPF [4]. In addition, multiple wound repair signaling pathways, such as the coagulation cascade, are dysregulated in IPF [4].

While a number of IPF biomarkers have been discovered, their diagnostic and predictive abilities lack sensitivity and specificity due to the heterogenic nature of the disease [4, 5]. Given the current progress in next‐generation sequencing and mass spectrometry, considerable attention has been paid to omics approaches and biomarker discovery for understanding this heterogeneous disease. Despite accumulating genomic, transcriptomic, and proteomic data for IPF, evidence from metabolomics has been minimal [6].

Among the various omics, metabolomics is based on the global profiling of metabolites in a biological system [7, 8] and it is broadly acknowledged to be the omics closest to the phenotype [9]. Metabolic profiling of biofluids and tissues provides information on changes in the abundance of endogenous metabolites associated with cellular responses to disease. One of the major advantages of metabolomics is that there is great similarity among species [10]. Thus, metabolomics allows us to discuss the direct connection between mouse and human.

Lipidomics has revealed how lipids, such as prostaglandin E_2_, take part in the pathophysiological molecular mechanism of IPF development [11, 12, 13], and has led to the identification of several candidate biomarkers for IPF [14]. Although several water‐soluble metabolites are significantly altered in the lungs of IPF patients compared with the healthy controls [7], it remains poorly understood whether water‐soluble metabolites could be useful biomarkers for IPF and how water‐soluble metabolites are involved in the pathophysiology of IPF development.

Here, we report the results of gas chromatography/mass spectrometry (GC/MS)‐based water‐soluble metabolomics in bronchoalveolar lavage fluid (BALF) and serum samples derived from pulmonary fibrosis model mice treated with bleomycin in an effort to identify novel IPF biomarker candidates. Furthermore, after analyzing a public RNA‐Seq database of IPF patient lungs, we report changes in gene expression related to water‐soluble metabolites.

## Materials and methods

### Bleomycin‐induced pulmonary fibrosis model mice

Sex‐, age‐, and weight‐matched C57BL/6J mice (8–10 weeks of age) were anesthetized using 13 μL·g^−1^ of 4% tribromoethanol. A maximal 1‐cm midline cervical incision was made to expose the trachea, followed by intratracheal instillation of PBS as a vehicle or 1.2 U·kg^−1^ of bleomycin (Nippon Kayaku, Tokyo, Japan). The cervical incision was closed with *n*‐butyl cyanoacrylate (Vetbond, 3M Health Care, St. Paul, MN, USA), and the mice were returned to their cages to recover. Mice were sacrificed 7 days after instillation to obtain BALF and serum samples. All experimental procedures were performed according to guidelines of the Committee on Ethical Use of Laboratory Animals of the Osaka University Medical Department.

### Azan staining and Ashcroft scoring

The lung samples were processed and then stained with the Azan stain as previously described [15]. Fibrosis was quantitatively evaluated from the light microscope images (×20 magnification) of the stained tissue sections by Ashcroft scoring [16]. To assess the severity of the fibrosis in the lungs (independent of inflammation), 10 fields/slide were evaluated. Each field was assigned a score between 0 (healthy lung) and 8 (total fibrosis). The average score of the 10 fields was defined as the Ashcroft score of the corresponding tissue section.

### Collection of BALF and serum samples

The trachea of each animal was surgically exposed and intubated with a syringe catheter. The lungs underwent lavage with 1 mL prewarmed PBS three times. After that, the cells in BALF were pelleted by centrifugation at 500 ***g*** for 10 min, and the supernatant was collected as BALF. Blood samples were collected independently from mice treated with PBS or bleomycin through cardiac puncture under the appropriate anesthesia and centrifuged to isolate animal serum; the supernatants (serum samples) were used for subsequent analyses. All mice were immediately euthanized using carbon dioxide according to the standard protocol of our animal facility. Four mice per group were utilized for the preparation of BALF and serum samples. We referred to a previous report by Tsujino *et al*. [17] to decide on the sample collection methods and the number of animals.

### Sample preparation for GC/MS analysis

Bronchoalveolar lavage fluid samples (1 mL) were placed in 2‐mL Eppendorf tubes, frozen with liquid nitrogen, and dried using a lyophilizer. Dried samples were extracted with 500 µL of extraction solvent consisting of 2.5 : 1 : 1 (v/v/v) methanol, distilled water, and chloroform, and then vortexed for 20 s. Serum samples (50 µL) were added to 450 µL of extraction solvent composed of 5.6 : 1.2 : 2.2 (v/v/v) methanol, distilled water (considered as serum), and chloroform, and then vortexed for 20 s. The internal standard, composed of 0.4 mg·mL^−1^ ribitol and 0.4 mg·mL^−1^ 10‐camphorsulfonic acid, was then added. Subsequently, the mixtures were incubated for 30 min at 37 °C before centrifuging at 16 000 ***g*** for 5 min at 4 °C. Aliquots of the supernatants (BALF, 400 μL; serum, 450 µL) were transferred to clean 1.5‐mL Eppendorf tubes, and distilled water was added (BALF, 200 μL; serum, 400 µL). After mixing, the solutions were centrifuged at 16 000 ***g*** for 5 min at 4 °C and the supernatants (BALF, 300 μL; serum, 400 µL) were dispensed into fresh 1.5‐mL Eppendorf tubes and capped. The extracts were evaporated using a vacuum centrifuge dryer for 2 h and finally lyophilized overnight.

For oximation, 100 μL of methoxyamine hydrochloride in pyridine (20 mg·mL^−1^) was added to the lyophilized samples and the samples were incubated for 90 min at 30 °C. For trimethylsilylation, 50 μL of *N*‐methyl‐*N*‐(trimethylsilyl)trifluoroacetamide was added to the samples and the samples were incubated for 30 min at 37 °C. Subsequently, the samples were centrifuged at 16 000 ***g*** for 5 min at 20 °C; 1 μL aliquots of the resultant supernatant were injected into the GC/MS.

### GC/MS analysis

Samples were analyzed using an AOC‐20is series injector (Shimadzu, Tokyo, Japan), a GC‐2010 Plus (Shimadzu), and a GCMS‐QP2010 Ultra (Shimadzu). Both platforms utilized a 30 m × 0.25 mm i.d. fused silica capillary column coated with 0.25 μm CP‐SIL 8 CB Low Bleed/MS (Agilent Technology, Santa Clara, CA, USA). The front inlet temperature was 230 °C, and the helium gas flow rate through the column was 1.12 mL·min^−1^. The column temperature was held at 80 °C for 2 min, isothermally, and then raised by 15 °C·min^−1^ to 330 °C and maintained for 6 min, isothermally. The transfer line and ion source temperatures were 250 and 200 °C, respectively. Scans were recorded at a rate of 20 scans·s^−1^ over a mass range of 85–500 *m/z*.

### Data processing and metabolite annotation

MS data were exported in the ANDI format. Peak detection and alignment were performed using the metalign software (Wageningen University, The Netherlands, freely available at https://www.wur.nl/en/show/MetAlign‐1.htm) [18]. Metabolites were annotated using aioutput2 (version 1.01) [19, 20] based on comparison of each MS spectrum with an in‐house library prepared from authentic standard chemicals [21].

### RNA‐Seq analysis of public data

To investigate the expression profile of pulmonary fibrosis‐related genes, we obtained public RNA‐Seq data (GSE92592) of lung tissues from IPF patients (*n* = 20) and healthy controls (*n* = 19) from NCBI GEO (https://www.ncbi.nlm.nih.gov/geo/). The data quality of the fastq files was verified with the fastqc tool (http://www.bioinformatics.babraham.ac.uk/projects/fastqc/). Read trimming was performed by trimmomatic version 0.36 (http://www.usadellab.org/cms/?page=trimmomatic) [22] with the Illumina Truseq adapter removal process (2 : 30 : 10) and the following options: LEADING:20, TRAILING:20, SLIDINGWINDOW:4:20, and MINLEN:25. Trimmed reads were mapped to the reference human genome, GRCh38, available in the Ensembl genome database (https://asia.ensembl.org/Homo_sapiens/Info/Index) using star program version 2.7.0b (https://github.com/alexdobin/STAR) [23] with mismatch option ‐‐outFilterMismatchNmax 2. RNA‐Seq by expectation‐maximization software version 1.3.0 (https://deweylab.github.io/RSEM) [24] was used for calculating the expression values in transcripts per million (TPM). Differentially expressed genes were identified using a cutoff false discovery rate of < 0.05. The false discovery rate was calculated by Storey's method [25] using qvalue package version 2.16.0 on r environment version 3.6.0 (https://www.r‐project.org/).

### Bioinformatics analyses

In the metabolome analysis, the signal intensities relative to the internal standard ribitol were used for downstream analyses. In the RNA‐Seq analysis, genes expressed in at least one sample were considered for downstream analysis, and the TPM values were converted to log2(TPM + 1). Principal component analysis (PCA) and heatmap generation were performed by the prcomp function and pheatmap package version 1.0.12, respectively. Disease enrichment analysis was performed by DOSE package version 3.10.2 and then visualized by enrichplot package version 1.4.0. All analyses described above were performed using r environment version 3.6.0. The metabolic network analysis was performed using metacore software version 19.4 (Clarivate Analytics, Philadelphia, PA, USA). Among the annotated metabolites, only those having significant variations were used as the input data for the metabolic network analysis.

### Statistical analyses

In the metabolome analysis, statistical significance was determined by Welch's *t*‐test using r environment version 3.6.0. *P* values < 0.05 were considered to be significant. Statistical analyses of metabolic networks were automatically calculated by a default method in metacore.

## Results and Discussion

### Metabolomic profiling analyses

**Fig. 1:**
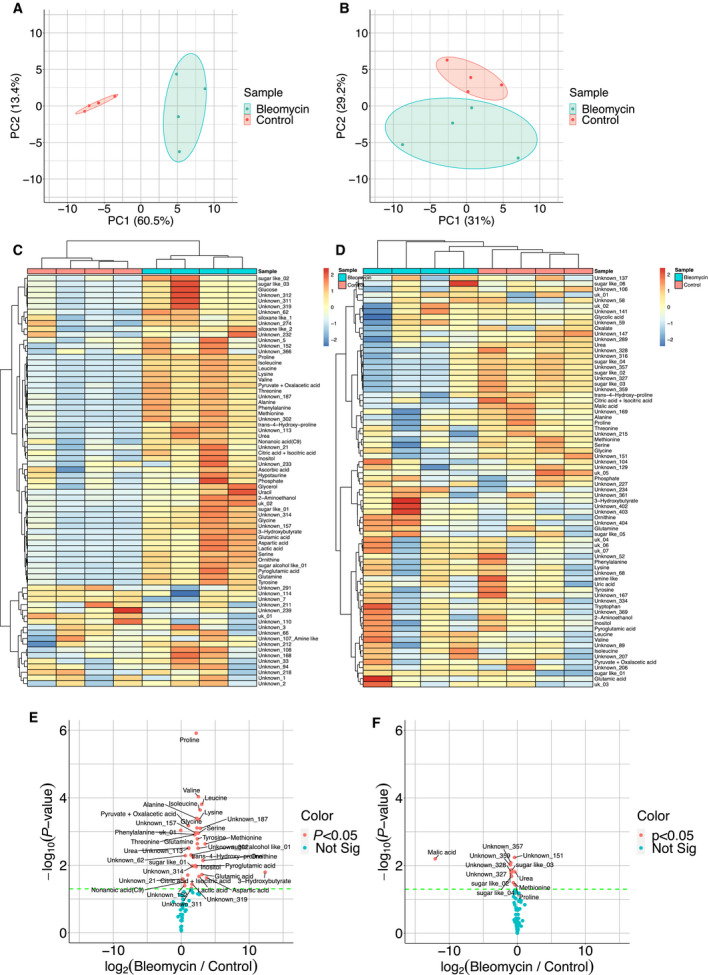
Characterization of metabolomic data. PCAs, heatmap, and volcano plots for metabolomic data of BALF (*n* = 4; A, C, E) and serum (*n* = 4; B, D, F) derived from lung tissue of pulmonary fibrosis model mice. In the heatmaps, hierarchical clustering was performed by the ward.D2 method. Each colored bar indicates the *z*‐score. In the volcano plots, significant metabolites were determined by Welch's *t*‐test with a threshold of *P* < 0.05. The green line indicates the threshold. Not Sig, not significant. The control group mice were treated with only PBS.

Azan staining and Ashcroft scoring showed that the average Ashcroft scores of the control and bleomycin groups were 0 and 3.55, respectively. These results indicate that the bleomycin‐induced pulmonary fibrotic mice remarkably exhibited lung fibrosis (Fig. S1A,B). The BALF and serum samples used in this study were obtained from four mice treated with or without bleomycin. Of the 73 water‐soluble metabolites detected in BALF, 32 were annotated. For serum, 29 of the 74 detected molecules were annotated. Notably, clusters between control‐ and bleomycin‐ groups were clearly separated by the PC1 axis in BALF and by the PC2 axis in serum. For PCA, the first principal component (PC1) described 60.5% of the variability in BALF and 31% of the variability in serum, while the second principal component (PC2) described 13.4% of the variability in BALF and 29.2% of the variability in serum (Fig. 1A,B). As expected, separation of BALF was more remarkable than that of serum. Collectively, metabolites, by our strategy, could discriminate bleomycin‐treated model mice from control mice.

While most metabolites in BALF were increased in response to bleomycin treatment, different results were observed in serum samples (Fig. 1C,D). In the volcano plot, 37 water‐soluble metabolites (25 annotated) were increased in response to bleomycin treatment in BALF, whereas 12 water‐soluble metabolites (4 annotated) were decreased in serum (Fig. 1E,F). The metabolomic profile of serum was heterogeneous, whereas that of BALF was homogeneous. Metabolites known to be upregulated in the lungs of IPF patients [7] such as glycine, glutamic acid, proline, and 4‐hydroxy‐l‐proline, were significantly upregulated in BALF of the mouse model (Fig. 1C,E). The metabolic profile positively correlates between BALF of mice and humans [26]. The results of the present study suggest that the metabolic profile of BALF from the model mice is similar to that of BALF from IPF patient lungs. Of interest, proline, methionine, and urea were increased in BALF, whereas they were decreased in serum (Fig. 1C–F). Thus, BALF was more useful than serum for discovering water‐soluble metabolites as IPF biomarkers.

### Metabolic network analysis shows the metabolic events in the fibrotic lung

**Fig. 2:**
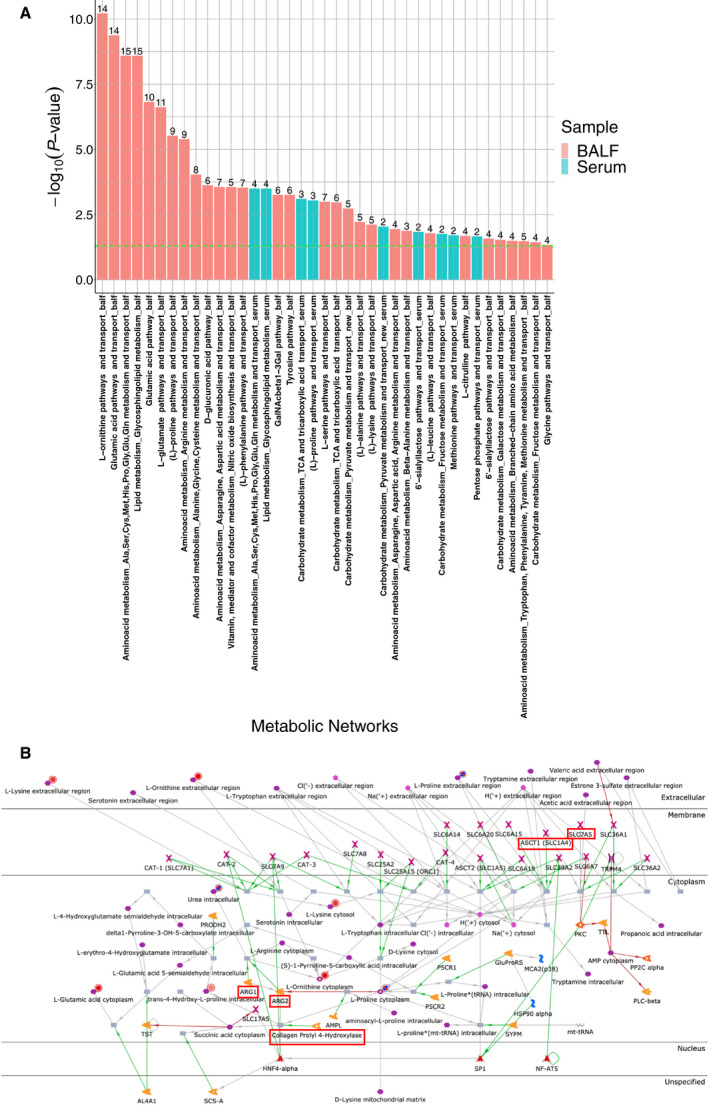
Metabolic network analyses using significantly changed metabolites in BALF and serum. (A) Enriched metabolic networks in BALF and serum. The green line indicates the threshold (*P* = 0.05). The number above each bar indicates the number of mapped metabolites in the network. (B) (l)‐Proline pathways and transport in metacore version 19.4. The green, red, and gray arrows indicate positive effect, negative effect, and unspecified effect, respectively. Closed red circles or mixed red/blue circles indicate differentially changed metabolites in BALF or serum samples from fibrotic lung mice, while red squares indicate proline‐ or methionine‐related genes. Further explanations are provided at https://portal.genego.com/legends/MetaCoreQuickReferenceGuide.pdf.

To understand the global dynamics in metabolism, we performed a metabolic network analysis. While 30 networks were enriched in BALF, only nine were enriched in serum (Fig. 2A). Of note, seven networks overlapped between BALF and serum (Table 1). While amino acid metabolism‐ and transport‐related networks were often enriched in BALF, several carbohydrate metabolism‐related networks were enriched in both BALF and serum (Fig. 2A and Table 1). The metabolic network involving (l)‐proline pathways and transport was enriched in BALF and serum (Fig. 2A). Proline, ornithine, and *trans*‐4‐hydroxy‐proline were mapped on the network (Fig. 2B). The network and publications cited in the database showed that (a) SLC1A4 is a transporter that moves proline into cells [27], (b) collagen prolyl 4‐hydroxylase (P4H) catalyzes the conversion of proline to *trans*‐4‐hydroxy‐proline [28], and (c) ornithine and urea are generated through the hydrolysis of arginine by arginase 2 (ARG2) [29]. In addition, ornithine aminotransferase (OAT) converts ornithine into proline; its gene expression is increased in the lung tissue of IPF patients compared with that of healthy controls [7], and SLC7A5 is a transporter that moves methionine into cells [30].

**Table 1:** Metabolic networks enriched in BALF and serum.

Metabolic network	BALF		Serum	
	*P*‐value	In data	*P*‐value	In data
Lipid metabolism_Glycosphingolipid metabolism	2.56E‐09	5	0.000318	4
Aminoacid metabolism_Ala,Ser,Cys,Met,His,Pro,Gly,Glu,Gln metabolism and transport	2.56E‐09	5	0.000318	4
(l)‐Proline pathways and transport	2.98E‐06	9	0.000909	3
Carbohydrate metabolism_TCA and tricarboxylic acid transport	0.00108	6	0.00079	3
Carbohydrate metabolism_Pyruvate metabolism and transport_new	0.00186	5	0.0091	2
6′‐sialyllactose pathways and transport	0.0266	4	0.0145	2
Carbohydrate metabolism_Fructose metabolism and transport	0.0362	4	0.0174	2

### Public RNA‐Seq data of IPF lungs reveal the expression profiles of proline‐ and methionine‐related genes

**Fig. 3:**
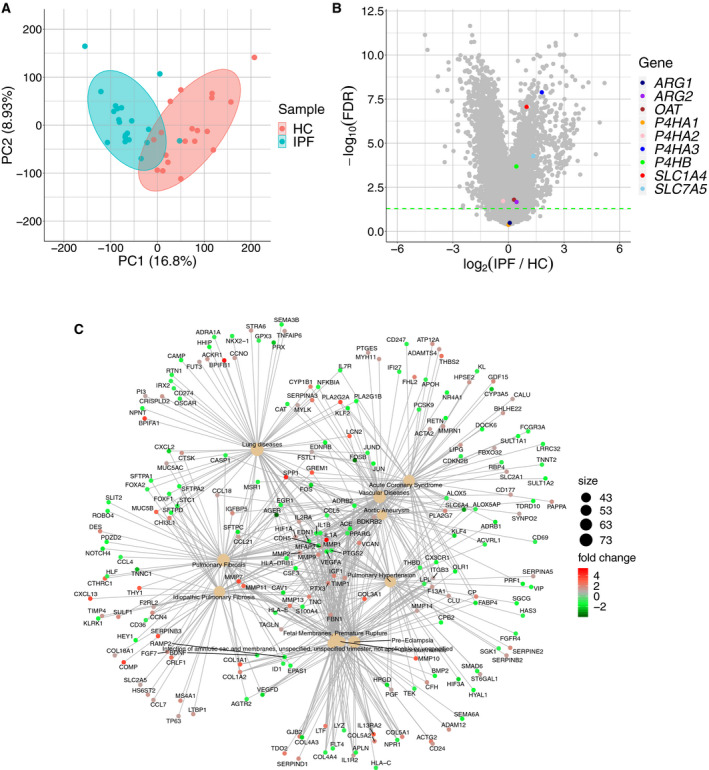
RNA‐Seq analysis of IPF and healthy control lung tissue using a public database. (A) PCAs. The input data comprised genes expressed in at least one sample; each dot indicates an individual patient. (B) Disease enrichment analysis. Colored circles indicate log_2_ (fold change). The circle size of each disease term indicates the number of genes connected to the term. The heat color indicates the fold change value of each gene. (C) Volcano plot. Differentially expressed genes were determined by Storey's method with a threshold of false discovery rate (FDR) < 0.05. The green line indicates the threshold. HC, healthy control.

To investigate the expression profiles of genes encoding the enzymes described above, we analyzed public RNA‐Seq data of lung tissue from IPF patients. Using PCA, clusters of control and IPF patients separated along the PC1 axis (Fig. 3A). Disease enrichment analyses showed that IPF, pulmonary fibrosis, and lung diseases were highly enriched (Fig. 3B), indicating that the features of IPF at the transcript level were included in the data (Fig. 3B). The volcano plot showed that the expressions of *SLC1A4*, *SLC7A5*, *ARG2*, *OAT*, *P4HA3,* and *P4HB* were increased significantly in the lung tissue of IPF patients (Fig. 3C). Thus, we speculate that the transport and metabolism of differentially expressed metabolites may be altered in fibrotic lung tissue.

The lungs of IPF patients are exposed to potent oxidative stress [31]. Indeed, the level of oxidized methionine is increased in BALF of IPF patients compared with that in healthy controls [32]. Because methionine plays a role in diminishing oxidative stress by auto‐oxidation [33], it might be transported from blood to the lungs to decrease oxidative stress caused by fibrosis. Proline and hydroxyproline are major components of collagen that are necessary for repairing injured lungs [34]. Thus, proline might be transported from blood to the lungs to support collagen synthesis for injury repair.

We analyzed water‐soluble metabolomic data of BALF and serum derived from mouse fibrotic lung tissue. Interestingly, proline, methionine, and urea were increased in BALF, whereas they were decreased in serum. Previous reports showed that proline, hydroxyproline, valine, leucine, isoleucine, alanine, and phenylalanine were elevated in exhaled breath of IPF patients [35]. These metabolites were also elevated in BALF of our mouse model. This result suggests that exhaled breath could partially reflect the metabolite signature in BALF. Moreover, RNA‐Seq data showed that transporters that take part in the uptake of these metabolites were increased significantly in the lungs of IPF patients. Based on these results, we believe that these metabolites may be transported from the blood to the lungs to repair the injury or to ameliorate oxidative stress caused by bleomycin treatment.

In this study, we analyzed two single‐layer omics independently. Because single‐layer omics are not sufficient to discover novel biomarkers [36], we need to integrate additional omics layers, such as the proteome, transcriptome, and genome. Therefore, in the future we will analyze human BALF, serum samples, and exhaled breath samples using multi‐omics and validate our findings using *in vitro* or *in vivo* assay systems.

## Conclusions

In this study, we found that proline and methionine levels were significantly increased in BALF derived from pulmonary fibrosis model mice treated with bleomycin, whereas they were significantly decreased in serum samples compared to those in the control. These results suggest that proline and methionine in BALF may be potential biomarkers for lung fibrosis. In addition, the expression of proline‐ and methionine‐related genes encoding transporter or converting enzymes, *SLC1A4*, *SLC7A5*, *ARG2*, *OAT*, *P4HA3*, and *P4HB*, was significantly increased in the lungs of patients with IPF compared to that in the lungs of healthy controls. Finally, we presented a potential mechanism underlying the pathogenesis of lung fibrosis. We believe that proline and methionine are transported from the blood to the lungs and their metabolism may contribute to fibrotic lung progression via inducing the expression of proline‐ and methionine‐related genes encoding their transporter or converting enzymes.

## Conflict of interest

The authors declare no conflict of interest.

## Author contributions

YN and YT conceived and designed the experiments. YN, YM, TB, EF, and YT performed the experiments and analyzed the data. YN and YT contributed to the writing of the paper under draft version. All authors discussed data and helped with manuscript preparation. All authors read and approved the final manuscript.

## Supporting information


**Fig. S1.** Azan staining and Ashcroft scoring of the lung from bleomycin‐induced pulmonary fibrotic mice. A; Azan staining of the lung from the fibrotic mice. Bars indicate 100 μm. B; Ashcroft scores were 6 evaluated using the images in Fig. S1‐A, as described in Materials and Methods section. Error bar 7 indicates standard deviation (n = 4). Statistical significance was determined by Welch's t‐test.

## Data Availability

All data supporting the findings of this manuscript are available from the corresponding authors upon reasonable request. The raw data of GSE92592 dataset are available from NCBI GEO (https://www.ncbi.nlm.nih.gov/geo/).
