# Supplementary material for: Metabolomic analysis of fibrotic mice combined with public RNA‐Seq human lung data reveal potential diagnostic biomarker candidates for lung fibrosis
Source: FEBS Open Bio. 2020 Oct 5;10(11):2427–36. doi: 10.1002/2211-5463.12982 (PMC7609803; doi:10.1002/2211-5463.12982)
Supplement: Supplementary file 1 — Fig. S1. Azan staining and Ashcroft scoring of the lung from bleomycin‐induced pulmonary fibrotic mice. A; Azan staining of the lung from the fibrotic mice. Bars indicate 100 μm. B; Ashcroft scores were 6 evaluated using the images in Fig. S1‐A, as described in Materials and Methods section. Error bar 7 indicates standard deviation (n = 4). Statistical significance was determined by Welch's t‐test. [file FEB4-10-2427-s001.pdf]

Supporting information

Supplementary figure legend

Fig. S1. Azan staining and Ashcroft scoring of the lung from bleomycin-induced pulmonary fibrotic mice.

A; Azan staining of the lung from the fibrotic mice. Bars indicate 100 μm. B; Ashcroft scores were evaluated using the images in Fig. S1-A, as described in Materials and Methods section. Error bar indicates standard deviation (n = 4). Statistical significance was determined by Welch's *t*-test.

Fig. S1

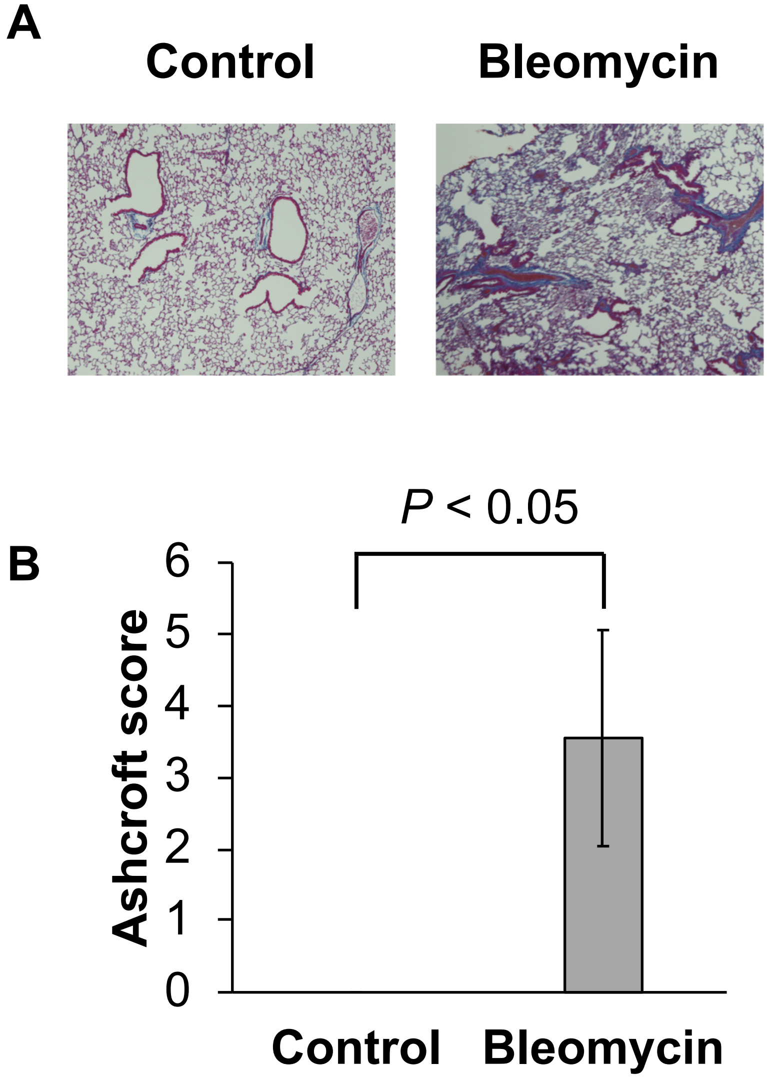
A
Control
Bleomycin
B
P < 0.05
6
5
4
3
2
1
0
Ashcroft score
Control
Bleomycin
